# State responsiveness, collective efficacy and threat perception: Catalyst and complacency effects in opposition to crime across eight countries

**DOI:** 10.1111/bjso.12832

**Published:** 2024-12-11

**Authors:** Chanki Moon, Giovanni A. Travaglino, Alberto Mirisola, Pascal Burgmer, Silvana D'Ottone, Isabella Giammusso, Hirotaka Imada, Kengo Nawata, Miki Ozeki

**Affiliations:** ^1^ Institute for the Study of Power, Crime and Society, Department of law and Criminology Royal Holloway University of London Egham UK; ^2^ Department of Psychology, Educational Science and Human Movement University of Palermo Palermo Italy; ^3^ School of Psychology University of Southampton Southampton UK; ^4^ School of Psychology Pontificia Universidad Católica de Chile Santiago Chile; ^5^ Department of Psychology Royal Holloway University of London Egham UK; ^6^ Faculty of Humanities Fukuoka University Fukuoka Japan; ^7^ Faculty of Humanities and Social Sciences Okayama University Okayama Japan

**Keywords:** collective efficacy, community, organized crime, perceived threat, state responsiveness

## Abstract

Collective action can be a crucial tool for enabling individuals to combat crime in their communities. In this research, we investigated individuals' intentions to mobilize against organized crime, a particularly impactful form of crime characterized by its exercises of power over territories and communities. We focused on individuals' views and perceptions of state authorities, examining how these views may be linked to intentions for collective mobilization. Using a large dataset with participants from eight countries (*N*_Total_ = 2088), we tested two distinct and opposing indirect paths through which perceived state responsiveness may be associated with collective mobilization intentions against organized crime, namely increased collective community efficacy (a Catalyst Indirect Effect) and diminished perceived threat from criminal groups (a Complacency Indirect Effects). Results showed that state responsiveness was associated with stronger collective action intentions through increased collective community efficacy. There was also some evidence of reduced collective action intentions through diminished perceived threat. These findings highlight the complex role of state responsiveness in predicting people's intentions to mobilize against collective problems in their communities. Implications of the findings, limitations and future directions are discussed.

## INTRODUCTION

The presence of organized criminal groups such as gangs, mafias and other illegal networks has posed a longstanding challenge to communities globally (von Lampe, 2016). These groups constitute a substantial threat worldwide due to their use of violence, their capacity to control territories and their ability to establish alternative systems of power and governance that operate in parallel to and in direct defiance of state authority (Travaglino & Abrams, 2019; von Lampe, 2016). Given its capacity to affect entire territories and subvert state authority, an active and engaged civil society is crucial in the fight against organized crime. Indeed, existing literature suggests that engagement in collective action is a key mechanism through which individuals and communities can achieve social change and enhance their standing across various domains and issues (Abrams & Grant, 2012; van Zomeren, 2016), including reactions to crime (Matsueda, 2006; Schreurs et al., 2019). In areas affected by crime, civil society's collective mobilization can serve as an important deterrent, empowering community members to restore safety in their environments.

Yet, despite the significant threat criminal organizations pose to societal stability and security, the predictors of collective mobilization against such entities remain underexplored. Prior studies have principally focused on the Southern Italian context and addressed the cultural values that may inhibit collective opposition against organized crime (e.g., Travaglino et al., 2014). In this research, we leveraged a large multinational survey to test hypotheses concerning individuals' views of state authorities. We drew on evidence indicating that perceptions of state responsiveness, namely the belief that state authorities are attentive to public needs, play a pivotal role in motivating citizens' engagement across various contexts and societal challenges (Sjoberg et al., 2015; Smets & Van Ham, 2013). We extended this work by investigating individuals' beliefs about state responsiveness in the context of communities' collective intentions against organized crime.

**FIGURE 1:**
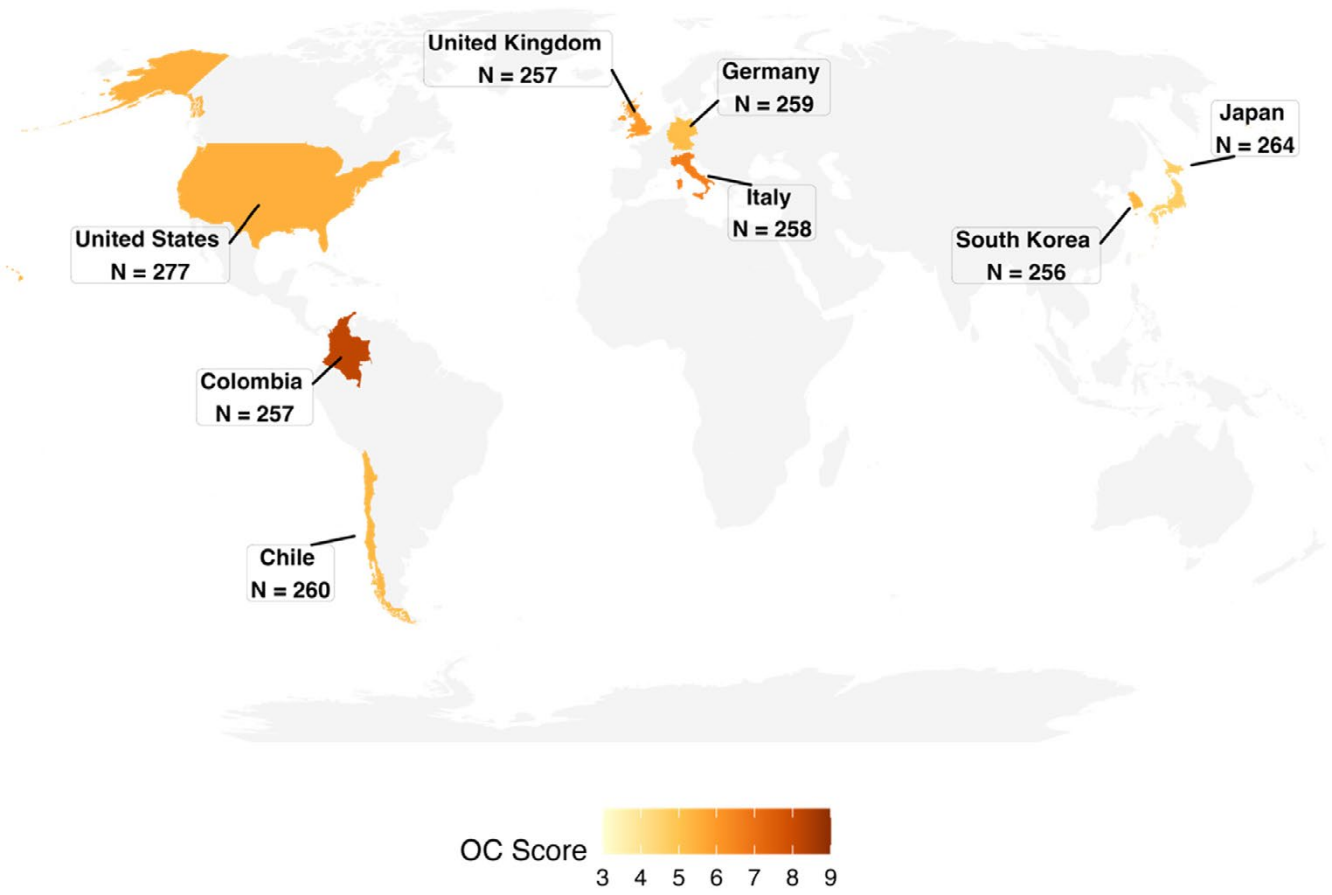
Sample size for the countries included in the study. OC Score = Organized Crime Score, referring to the extent to which a country is affected by the presence of organized criminal groups (Global Initiative, 2023).

We investigated two potential psychological pathways through which beliefs about state responsiveness might be linked to individuals' intentions to oppose organized crime collectively. Specifically, we hypothesized that state responsiveness would positively predict individuals' intention to engage in collective action against organized criminal groups through a heightened sense of collective community efficacy. Simultaneously, however, we also expected that state responsiveness would be linked to a diminished sense of threat from organized crime, thus yielding a negative indirect association with collective engagement. We tested our hypotheses using a dataset comprising respondents from eight nations (see Figure 1), including countries from Europe (the UK, Italy, Germany), East Asia (South Korea, Japan), North (the US) and South America (Chile and Colombia). All the countries surveyed are affected by the presence of organized crime, although the extent of such presence varies across nations (Global Initiative, 2023; see also Travaglino et al., 2024). Thus, we tested our hypotheses using data from participants living in a heterogeneous set of different contexts.

### Collective action and state responsiveness in the context of crime

In broad terms, citizens can tackle crime‐related challenges either by delegating action to institutions or through direct collective engagement (Matsueda, 2006). Institutional action is characterized by routine, formality and strict adherence to rules. This approach commonly involves formal organizations, such as central and local governments and police agencies and is closely linked with the criminal justice system.

Conversely, collective action refers to citizen‐led initiatives that occur outside of institutional settings and within informal groups or assemblies (Matsueda, 2006; Sampson et al., 1997). Such actions can take various forms, from public protest and unrest (Useem, 1998) to participation in professional, educational and other voluntary associations (Welzel et al., 2005), including neighbourhood organizations (Mesch & Schwirian, 1996). Collective actions are more spontaneous, relying on the formation of grassroots mobilization (Matsueda, 2006). For instance, in the context of opposition to criminal organizations in Italy, several ‘anti‐mafia’ groups and associations have emerged. These groups' activities range from carrying out educational events in public schools to coordinating initiatives against racketeering to public demonstrations and protests (Jamieson, 1999; Schneider & Schneider, 2001; Travaglino et al., 2014).

The presence of an active civil society is especially relevant to contrast organized crime because criminal organizations can become deeply socially embedded. They can offer services (e.g., ‘protection’) and illegal goods to individuals while pursuing power, reputation and status in the community (Travaglino & Abrams, 2019; von Lampe, 2016). Thus, it is essential for citizens to maintain high public awareness about the presence of these groups and to cultivate social norms that increase the repercussions of dealing with them (Lavezzi, 2014).

Yet, most research on societal responses to crime has been conducted in the context of institutional action (cf. Matsueda, 2006). Relatively less research has examined individuals' collective intentions against these groups. For instance, Travaglino and colleagues have investigated the role of culturally rooted values in individuals' intentions to engage in collective anti‐mafia action in Southern Italy (Travaglino et al., 2014). They focussed on the role of individuals' endorsement of masculine honour in predicting lower mobilization intentions. In the present study, we extended this research by investigating a distinct set of factors that may be associated with individuals' intentions to oppose organized criminal groups. Specifically, we focused on the perception of state responsiveness, individuals' sense of collective community efficacy and perceived threat from organized crime.

State responsiveness refers to individuals' belief regarding the political system's attentiveness to their needs (Balch, 1974; Craig et al., 1990). Perceptions of state responsiveness are critical to mobilization because citizens' collective responses to crime or other societal challenges often depend on establishing a positive collaborative dynamic between citizens and institutions (Bullock & Sindall, 2014; Matsueda, 2006; Terpstra, 2010). On the one hand, formal institutions, including governments, seek to engage citizens more actively in the political process (Dutil et al., 2008; Yetano et al., 2010), recognizing the benefits such engagement brings to the quality of democratic governance and the political system overall. On the other, research on political participation has shown the existence of a positive link between state responsiveness (also labelled external political efficacy) and citizen engagement. This evidence suggests that the more responsive a government is perceived to be, the more likely its citizens are to actively participate in actions that benefit the collective (Sjoberg et al., 2015; Smets & Van Ham, 2013; Travaglino & Moon, 2020). For instance, in the area of crime prevention, individuals exhibit greater reluctance to report crime when perceiving lower state responsiveness (Wu et al., 2022).

Despite the enormous importance of individuals' views of state authorities in collective mobilization, relatively fewer social psychological studies have integrated this construct into models of collective action (e.g., Becker & Tausch, 2015; Corcoran et al., 2011; Gulevich et al., 2017; Heering et al., 2020; Tausch et al., 2011; Travaglino, 2019; Travaglino & Moon, 2020). In the present research, we posited that state responsiveness may be indirectly linked to collective action intentions against organized crime via two distinct and oppositional indirect paths. Specifically, we focussed on heightened collective community efficacy and a diminished sense of threat from organized crime. We hypothesized that while the path via heightened collective community efficacy may catalyse increased collective action (a Catalyst Indirect Effect), the path via reduced perceived threat from criminal groups may deter collective action by inducing a degree of complacency in individuals (a Complacency Indirect Effect).

### Collective community efficacy and a catalyst indirect effect

Research indicates that a sense of collective efficacy is an essential driver of people's motivation to engage in collective action (e.g., Hornsey et al., 2006, 2015; Van Zomeren et al., 2004, 2008). Collective efficacy refers to the shared belief that a group has the resources and skills needed to address a common problem (Van Zomeren et al., 2004, 2008) and the capacity to develop and execute the necessary actions to achieve their desired goals (Sampson et al., 1997). Collective efficacy is especially relevant in mobilizing individuals who would not otherwise label themselves as activists (Smith et al., 2021).

Within communities, a sense of collective efficacy fosters social cohesion and promotes engagement (Gearhart, 2019; Gearhart & Joseph, 2019; Maton, 2008). High levels of collective efficacy benefit a community in several ways. For instance, collective community efficacy contributes to the prevention of interpersonal violence in urban neighbourhoods, also after accounting for the demographic composition of the residents, markers of disadvantage and other neighbourhood characteristics (Sampson et al., 1997). Moreover, collective community efficacy plays an important role in community‐based crime prevention strategies (cf. Gearhart, 2023). Communities characterized by high collective efficacy tend to exhibit more effective mechanisms of social control and to experience fewer crime‐related issues (Gerell & Kronkvist, 2016; Mazerolle et al., 2010; Weisburd et al., 2020).

We tested the hypothesis that individuals' sense of collective community efficacy is positively predicted by the perceived responsiveness of political institutions. Evidence shows that when individuals perceive institutions as attentive to their needs and acting in the community's best interest, they feel empowered to take responsibility for their own safety (Lion et al., 2002; Paton, 2013, 2019; Poortinga & Pidgeon, 2004; Yesberg et al., 2023). Conversely, institutions perceived as distant and unresponsive have been linked to lower community efficacy because individuals do not feel they have an adequate level of support to guide their actions (Paton, 2013). Thus, we expected that state responsiveness would be positively associated with collective community efficacy, which, in turn, should predict stronger intentions to engage in collective action against criminal groups.

### Perceived threat and a complacency indirect effect

An additional factor likely to play a role in individuals' collective intentions against organized crime is threat appraisal (cf. Oh & Kim, 2009; Vuori et al., 2013). Threat appraisal refers to the perception of organized crime as a significant societal issue that warrants collective engagement. We hypothesized that threat appraisals would be positively associated with collective intentions against organized crime.

Research in various domains corroborates the idea that perceiving a societal issue as a significant threat can motivate collective action. For instance, viewing climate change as a threat can encourage pro‐environmental behaviour and collective efforts to respond to the crisis (Amel et al., 2017; Stollberg & Jonas, 2021). Similarly, research has shown that, in the context of human‐caused earthquakes, individuals living where the objective threat of an earthquake was stronger were more likely to perceive these events as violations of their rights, prompting increased mobilization (Kutlaca et al., 2019).

In the context of terrorism, research has consistently shown that heightened perceived threats to security significantly influence public attitudes, leading to greater support for counterterrorism policies and even endorsements of civil liberty restrictions (Davis & Silver, 2004; Dietrich & Crabtree, 2019; Valentino et al., 2021). Moreover, during the COVID‐19 pandemic, the perceived threat from the virus was associated with stronger public willingness to accept state surveillance measures (e.g., Lalot et al., 2022; Wnuk et al., 2020).

Overall, this body of research highlights that perceptions of societal threat are linked to individuals' willingness to engage in action for the common, even at high personal costs. This evidence supports the argument that in the context of organized crime, perceived threats can be associated with stronger collective action intentions as individuals and communities strive to protect themselves and restore their sense of security.

An important question is how the perception of state responsiveness may predict individuals' threat appraisals. A possibility that we test in the present study is that beliefs about institutional authorities' responsiveness might mitigate the perceived threat from criminal groups because individuals may believe the problem may be successfully tackled via institutional action, making direct collective mobilization seem less necessary. For instance, there is evidence that individuals' confidence in institutions and their beliefs about institutions' performance are linked to lower levels of fear of crime (Lin, 2023), a construct linked to (albeit conceptually distinct from) that of perceived threat (Huddy et al., 2002). This reasoning implies that individuals' beliefs about state responsiveness should be negatively associated with threat, yielding an indirect complacency effect on mobilization.

## METHODS

### Participants and procedure

The present research involved the participation of 2088 individuals from eight different countries (see Figure 1). Participants were from the UK (*N* = 257; 126 male, 129 female, 2 unreported; *M*_age_ = 47.88, *SD*_age_ = 15.83), Italy (*N* = 258; 136 male, 120 male, 2 unreported; *M*_age_ = 48.63, *SD*_age_ = 16.09), Germany (*N* = 259; 126 male, 130 female, 3 unreported; *M*_age_ = 51.59, *SD*_age_ = 15.39), South Korea (*N* = 256; 166 male, 89 female, 1 unreported; *M*_age_ = 43.05, *SD*_age_ = 14.61), Japan (*N* = 264; 152 male, 111 female, 1 unreported; *M*_age_ = 50.57, *SD*_age_ = 16.47), the US (*N* = 277; 130 male, 135 male, 12 unreported; *M*_age_ = 46.18, *SD*_age_ = 16.65), Chile (*N* = 260; 144 male, 115 female, 1 unreported; *M*_age_ = 41.23, *SD*_age_ = 13.89) and Colombia (*N* = 257; 135 male, 121 female, 1 unreported; *M*_age_ = 39.35, *SD*_age_ = 12.86).

Participants were recruited via an international research panel company (MSI‐ACI; https://site.msi‐aci.com/) using the software Qualtrics. They completed a series of measures tapping into their perception of organized crime and other variables after being informed of the research objective, being provided information on research ethics and giving consent. In exchange for their participation, participants were compensated for their time. All measures used in the present study were initially developed in English and then translated into Italian, German, Korean, Japanese and Spanish using the back‐translation procedure following the guidelines by Brislin (1986). Data and analysis code for this study are available at the following link: https://osf.io/hb7yk/

### Measures

#### State responsiveness

State responsiveness was measured using three items used in prior research (Craig et al., 1990; Geurkink et al., 2020). These items were: ‘Politicians are not interested in what people like me think’, ‘Political parties are only interested in my vote, not in my opinion’, and ‘People like me don't have any say about what the government does’. The items were answered on a 7‐point scale (*1 = strongly disagree*, *7 = strongly agree*). Higher scores indicate higher levels of perceived state responsiveness (α_tot_ = .83, α_UK_ = .85, α_IT_ = .83, α_GER_ = .92, α_KOR_ = .64,^1^ α_JPN_ = .79, α_US_ = .89, α_CHIL_ = .80, α_COL_ = .82).

#### Collective community efficacy

Participants responded to a four‐item measure of collective community efficacy. They were first provided with a detailed definition of ‘community’. The instructions read, ‘In the following statements, we will refer to the term “community” as the group of people living in your local area, such as your neighbourhood. This includes people you interact with regularly, as well as those you may not know personally but share the same spaces (e.g., live in nearby streets, go to the same shops, etc.)’. The four items were: ‘My community has a strong voice in important decisions that affect us.’, ‘My community is able to influence the actions of those in positions of power.’, ‘My community has the power to shape the direction and priorities of our area’ and ‘My community is able to mobilise and organise effectively to achieve our goals’. Higher scores indicate higher levels of collective community efficacy (1 = *strongly disagree*, 7 = *strongly agree*; α_tot_ = .93, α_UK_ = .95, α_IT_ = .94, α_GER_ = .90, α_KOR_ = .91, α_JPN_ = .94, α_US_ = .93, α_CHIL_ = .92, α_COL_ = .90).

#### Perceived threat

Participants were asked about the perceived threat from organized crime in their country using three items. They were provided with a definition of organized crime, ‘In many countries, there exist organised groups that operate outside the bounds of the law. These groups may include but are not limited to mafias, cartels, gangs, and other similar organisations that are not recognised by the government and do not comply with established legal frameworks. For the purpose of this scale, we refer to these groups as “criminal groups”’. The three items were as follows: ‘To what extent do you think that the presence of criminal groups is a concern in [Country]?’, ‘How much of a danger to public security are criminal groups in [Country]?’ and ‘How much of a danger to the economy are criminal groups in [Country]?’. The items were answered on a 7‐point scale (*1 = not at all*, *7 = a great deal*). Higher scores indicate higher levels of perceived threat from criminal groups (α_tot_ = .92, α_UK_ = .91, α_IT_ = .91, α_GER_ = .92, α_KOR_ = .90, α_JPN_ = .92, α_US_ = .92, α_CHIL_ = .80, α_COL_ = .80).

#### Collective mobilization against organized crime

Participants indicated their intention to engage in collective action against criminal groups using three items. Participants were asked about how likely they were to ‘Participate in a public protest against criminal groups’, ‘Become part of an association against criminal groups’ and ‘Convince other people to become part of an association against criminal groups’ (Travaglino et al., 2014). The items were answered on a 7‐point scale (1 = *Extremely unlikely*, 7 = *Extremely likely*). Higher scores indicate higher levels of intention to engage in collective action against criminal groups (α_tot_ = .87, α_UK_ = .89, α_IT_ = .89, α_GER_ = .83, α_KOR_ = .91, α_JPN_ = .90, α_US_ = .93, α_CHIL_ = .81, α_COL_ = .78).

#### Control variables

In our model, we included gender, age, subjective social status and political orientation to control for the potential impact of these variables. Participants used a 10‐point scale (1 = the bottom of the ladder, 10 = the top of the ladder) to rate their subjective social status based on the image of a ladder and the following instruction (Adler et al., 2000): ‘Think of the ladder above as representing where people stand in society. At the top of the ladder are the people who are best off—those who have the most money, most education and the best jobs. At the bottom are the people who are worst off—who have the least money, least education and the worst jobs or no job. The higher up you are on this ladder, the closer you are to people at the very top and the lower you are, the closer you are to the bottom. Where would you put yourself on the ladder?’ Participants also rated their political orientation using a single item (‘Considering the current political context in [Country], how would you describe yourself?’ answered on a scale of 1 to 10, where 1 = left‐winger and 10 = right‐winger).

### Data analytic strategy

To test our hypotheses, we analysed the data employing a structural equation model with latent variables. In this study, we accounted for individuals' clustering in different nations using fixed effects for countries (Huang, 2016; McNeish, 2023; McNeish & Kelley, 2019). Fixed effects control for and exclude all between‐cluster variability from the model, enabling us to focus on within‐cluster associations among variables. Because fixed effects in the model remove all cross‐cluster variability, they reduce the problem of omitted variable bias at the cluster level, such as institutional history or GDP (Huang, 2016). Results from models with fixed effects can be interpreted as average within‐cluster effects. Fixed effects are especially suitable for analysing data characterized by a relatively lower number of clusters (Bryan & Jenkins, 2016; Elff et al., 2021). Moreover, employing fixed effects enabled us to address the issue of small sample size within each cluster relative to the complexity of the model tested in the present study (Sim et al., 2022).^2^

The analysis was performed using the R software and the lavaan (Rosseel, 2012) package. Prior to testing our research model, we conducted a multi‐group confirmatory factor analysis to assess measurement invariance across the eight countries. Measurement invariance indicates that participants interpret the measurement scale similarly (Jeong & Lee, 2019; Milfont & Fischer, 2010). Invariance was assessed by comparing the difference in CFI (ΔCFI< .01) between nested models with constrained parameters (G. W. Cheung & Rensvold, 2002). The fit of the models was evaluated based on predetermined criteria: an acceptable fit was indicated by CFI ≥ .90, RMSEA ≤ .08 and SRMR < .10, while an excellent fit was suggested by CFI ≥ .95, RMSEA < .06 and SRMR < .08 (Chen et al., 2008; Gana & Broc, 2018; Hu & Bentler, 1998, 1999; Kline, 2016; Schumacker & Lomax, 2012).

## RESULTS

Means and standard deviations across nations are presented in Table 1. Zero‐order correlations across nations are presented in Table 2.

**TABLE 1:** Means and standard deviations across nations.

Variables	M (SD)								
	Total	UK	Italy	Germany	Korea	Japan	US	Chile	Colombia
Study variables									
SR	2.64 (1.45)	2.42 (1.33)	2.54 (1.32)	2.75 (1.62)	2.63 (1.18)	2.93 (1.37)	2.89 (1.63)	2.41 (1.40)	2.53 (1.56)
CCE	3.97 (1.51)	3.74 (1.44)	3.75 (1.54)	3.86 (1.42)	4.38 (1.30)	3.16 (1.39)	4.33 (1.48)	4.13 (1.56)	4.44 (1.49)
PT	5.36 (1.50)	4.85 (1.42)	6.20 (0.98)	5.05 (1.43)	4.29 (1.48)	4.76 (1.46)	4.99 (1.50)	6.27 (1.02)	6.47 (0.82)
CAI	3.11 (1.77)	2.53 (1.56)	3.83 (1.75)	2.64 (1.62)	3.75 (1.67)	2.15 (1.45)	3.32 (1.89)	3.47 (1.77)	3.23 (1.70)
Demographics									
Age	46.08 (15.81)	47.88 (15.83)	48.63 (16.09)	51.59 (15.39)	43.05 (14.67)	50.57 (16.47)	46.18 (16.65)	41.23 (13.89)	39.35 (12.86)
SSS	5.81 (1.76)	5.81 (1.86)	5.54 (1.62)	6.03 (1.75)	6.07 (1.77)	6.22 (1.79)	5.96 (1.93)	5.30 (1.54)	5.55 (1.56)
Political orientation	5.51 (2.31)	5.09 (2.07)	5.40 (2.65)	5.08 (1.78)	5.73 (1.93)	5.66 (1.71)	5.48 (2.69)	5.92 (2.71)	5.72 (2.57)

Abbreviations: CAI, collective action intention; CCE, collective community efficacy; PT, perceived threat; SR, state responsiveness; SSS, subjective social status.

**TABLE 2:** Correlations among study variables across nations.

	**Total**								**UK**							
	**1**	**2**	**3**	**4**	**5**	**6**	**7**	**8**	**1**	**2**	**3**	**4**	**5**	**6**	**7**	**8**
1. SR	–								–							
2. CCE	.22***	–							.30***	–						
3. PT	−.11***	.09***	–						.00	.11	–					
4. CAI	.04	.24***	.21***	–					.10	.27***	.19	–				
5. Gender	−.02	−.01	.07*	−.12***	–				−.01	.12	.01	−.02	–			
6. Age	.04	−.09***	.06	−.01	−.04	–			−.01	−.11	.12	−.12	−.08	–		
7. SSS	−.17***	−.19***	−.09***	−.14***	−.01	−.05	–		−.27***	−.27***	.04	−.13	−.02	−.13	–	
8. PO	−.04	.10***	.10***	.07*	−.04	.06	−.14***	–	.10	.12	.17	.04	−.09	.21*	−.20***	–
**Italy**									**Germany**							
	**1**	**2**	**3**	**4**	**5**	**6**	**7**	**8**	**1**	**2**	**3**	**4**	**5**	**6**	**7**	**8**
1. SR	–								–							
2. CCE	.21*	–							.34***	–						
3. PT	−.20*	−.07	–						−.29***	−.10	–					
4. CAI	−.05	.15	.24**	–					−.04	.13	.15	–				
5. Gender	.03	−.13	.07	−.16	–				−.07	−.10	.03	−.18	–			
6. Age	−.08	−.12	.22*	−.00	.00	–			.08	−.01	.13	−.03	−.03	–		
7. SSS	−.23**	−.29***	.10	−.17	.02	.05	–		−.20*	−.19	−.01	−.15	.07	.01	–	
8. PO	.02	.17	.07	−.07	−.03	.06	−.16	–	−.31***	.04	.19*	.06	−.05	.01	−.09	–
**Korea**									**Japan**							
	**1**	**2**	**3**	**4**	**5**	**6**	**7**	**8**	**1**	**2**	**3**	**4**	**5**	**6**	**7**	**8**
1. SR	–								–							
2. CCE	.32***	–							.23**	–						
3. PT	−.18	.08	–						−.07	.15	–					
4. CAI	.11	.17	.11	–					.11	.27***	.14	–				
5. Gender	−.15	−.01	.14	−.16	–				.02	.03	.06	−.12	–			
6. Age	−.04	−.10	.01	.19	−.19	–			.16	.09	.20*	.04	−.03	–		
7. SSS	−.15	−.07	.09	−.10	.11	−.09	–		−.18	−.11	−.14	−.01	−.20*	−.27***	–	
8. PO	.09	−.07	−.01	−.11	−.14	−.02	−.15	–	−.08	.03	.04	−.02	−.16	.03	.02	–
**US**									**Chile**							
	**1**	**2**	**3**	**4**	**5**	**6**	**7**	**8**	**1**	**2**	**3**	**4**	**5**	**6**	**7**	**8**
1. SR	–								–							
2. CCE	.29***	–							.21*	–						
3. PT	.09	.23**	–						−.02	.04	–					
4. CAI	.16	.41***	.30***	–					.04	.07	.26***	–				
5. Gender	−.01	−.01	.03	−.09	–				−.02	.01	.16	−.09	–			
6. Age	.05	−.09	.14	−.02	.02	–			.04	−.02	.29***	.23**	−.06	–		
7. SSS	−.19*	−.31***	−.09	−.23**	−.08	−.03	–		−.25**	−.13	.03	−.05	.07	−.13	–	
8. PO	−.01	.24***	.14	.24**	.00	.11	−.22**	–	−.03	.14	.24**	.13	−.02	−.01	−.05	–
**Colombia**																
	**1**	**2**	**3**	**4**	**5**	**6**	**7**	**8**								
1. SR	–															
2. CCE	.09	–														
3. PT	−.13	.14	–													
4. CAI	.02	.12	.06	–												
5. Gender	.02	.05	.07	−.15	–											
6. Age	−.02	.02	.16	.07	−.02	–										
7. SSS	−.06	−.05	−.05	−.11	.06	−.10	–									
8. PO	−.11	.00	.02	.10	.14	.25**	−.18	–								

Abbreviations: CAI, collective action intention; CCE, collective community efficacy; PT, perceived threat; SR, state responsiveness; SSS, subjective social status.

***p < .001; **p < .01; *p < .05.

### Measurement invariance

Our objective was to establish measurement invariance of the latent measures, including state responsiveness, collective community efficacy, perceived threat and collective action intention, across eight nations. Measurement invariance ensures that the measures are understood similarly across different national contexts, allowing for meaningful interpretation of the within‐country averages resulting from the model.

First, we estimated a configural‐invariance model for each of the four measures. The configural models for all the measures except the collective community efficacy measure had to be assumed because the models were just identified, RMSEAs = 0, CFIs = 1. The configural‐invariance model for the collective community efficacy measure had good fit (CFI = .996, RMSEA = .08), indicating a similar latent structure across countries. Next, we assessed metric invariance for the measures by constraining the factor loadings to be equal across nations. All the models achieved metric invariance: constraining the loadings to be equal across countries did not cause the measure models to deteriorate significantly (ΔCFIs ≤ .01). Full scalar invariance was achieved only for the measure of collective community efficacy (ΔCFI = .01). It was not attained for state responsiveness (ΔCFI = 0.04), perceived threat (ΔCFI = .02) and collective action intention (ΔCFI = .06). This indicates that the fit of these models deteriorated beyond the recommended threshold when the intercepts of the items were constrained to be equal across nations. By releasing the intercept of one item for each of the three measures (‘People like me don't have any say about what the government does’; ‘How much of a danger to the economy are criminal groups in [Country]?’; ‘Participate in a public protest against criminal groups’), we were able to achieve partial scalar invariance (ΔCFIs ≤ .01). Partial scalar invariance is often a more realistic goal for models involving multiple countries (Steinmetz, 2018).

### Confirmatory factor analyses and common method variance

We utilized MGCFA to verify discriminant validity among study variables used in the present structural equation model. The data were better represented by a four‐factor measurement model, *χ* ^2^(577) = 961.49, *p* < .001, CFI = .98, RMSEA = .05, SRMR = .05, as compared to other measurement models (3‐factor, 2‐factor and 1‐factor models; see Table 3). Furthermore, we conducted a Harman's single factor test to evaluate the presence of common method variance. Across countries, the factor accounted for only 29% (UK), 26% (IT), 25% (GER), 25% (KOR), 28% (JPN), 32% (US), 24% (CHIL) and 23% (COL) of total variance. This falls below the recommended 50% threshold (Podsakoff et al., 2003), suggesting that a single general factor does not account for most variance in the present model.

**TABLE 3:** Model fit results for multi‐group confirmatory factor analysis.

Models & structure	*χ* ^2^	*Df*	CFI	RMSEA	SRMR
4 factor model					
SR, CCE, PT, CAI	961.49	577	0.98	0.05	0.05
3 factor model					
[SR + CCE], PT, CAI	3387.18	615	0.82	0.14	0.12
[SR + PT], CCE, CAI	5755.10	615	0.68	0.19	0.18
[SR + CAI], CCE, PT	4745.37	615	0.74	0.16	0.16
[CCE + PT], SR, CAI	5773.57	615	0.68	0.18	0.18
[CCE + CAI], SR, PT	4421.40	615	0.76	0.16	0.14
[PT + CAI], SR, CCE	4614.04	615	0.75	0.16	0.15
2 factor model					
SR, [CCE + PT + CAI]	9445.64	645	0.38	0.24	0.22
CCE, [SR + PT + CAI]	7288.14	645	0.58	0.21	0.20
PT, [SR + CCE + CAI]	6867.58	645	0.60	0.20	0.18
CAI, [SR + CCE + PT]	8277.33	645	0.52	0.22	0.21
[SR + CCE], [PT + CAI]	7080.70	645	0.59	0.21	0.18
[SR + PT], [CCE + CAI]	9469.99	645	0.47	0.24	0.22
[SR + CAI], [CCE + PT]	9878.59	645	0.45	0.24	0.23
1 factor model					
[SR + CCE + PT + CAI]	11,913	667	0.31	0.26	0.24

Abbreviations: CAI, collective action intention; CCE, collective community efficacy; PT, perceived threat; SR, state responsiveness.

### Main analyses: Testing the catalyst and complacency indirect effects

**FIGURE 2:**
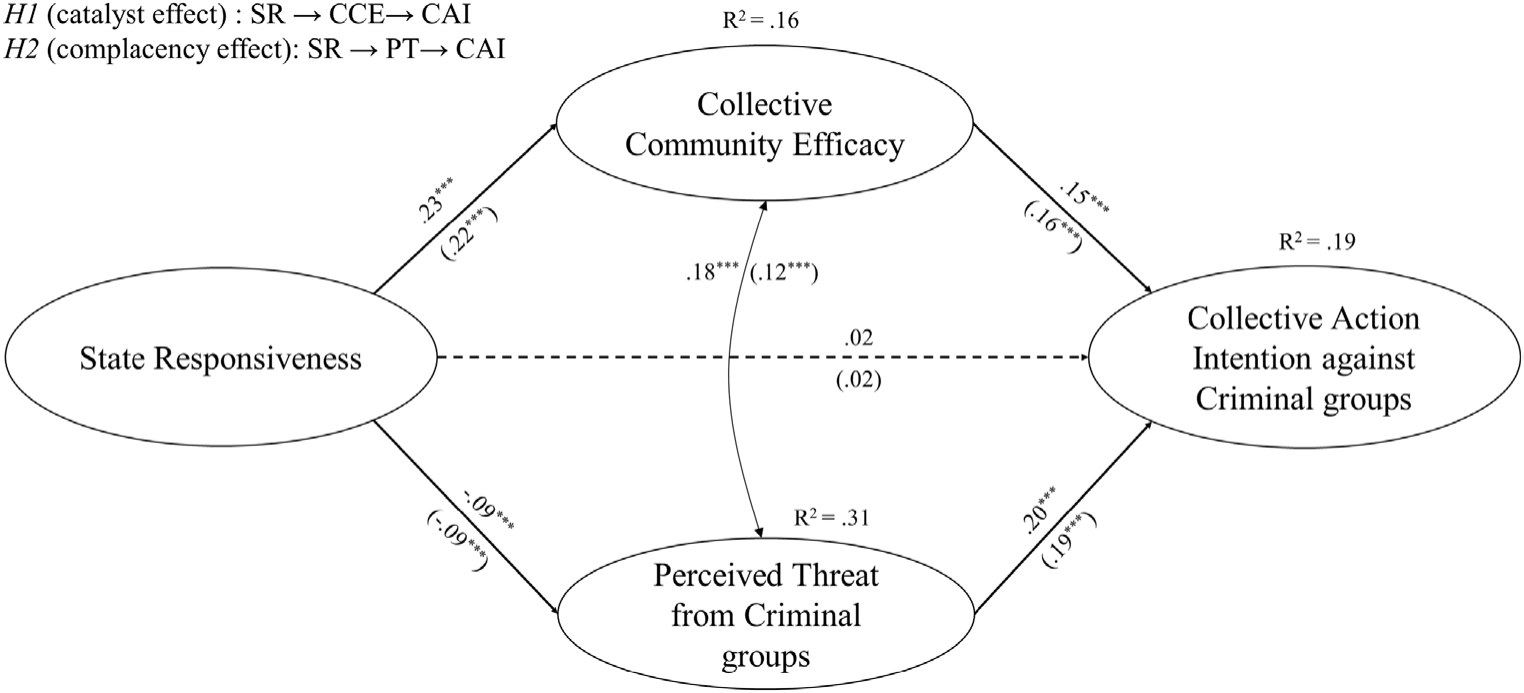
Structural equation model with country fixed effects. Standardized coefficients are presented in parentheses. Gender, age, subjective social status and political orientation were covariates in the model. ***p < .001.

We conducted a parallel indirect effect model to test our two hypothesized indirect effects, the *Catalyst* and *Complacency* indirect effects. The catalyst effect refers to the indirect pathway from state responsiveness to collective action intention through heightened collective community efficacy. The complacency effect is the indirect pathway from state responsiveness to collective action intention through diminished perceived threat. We included a correlational path between the two proposed mediating variables, collective community efficacy and perceived threat. Similar to previous research in the context of climate change (Hornsey et al., 2015), the perceived threat of organized crime was positively associated with collective efficacy. The model fit the data adequately, *𝜒* ^2^ (169) = 891.81, *p* < .001, CFI = .96, RMSEA = .05, SRMR = .04. Figure 2 summarizes the results.

The direct association between state responsiveness and collective action intention was not significant, *b* = .02, 95% CI [−.03, .07], *ꞵ* = .02, *p* = .537. However, individuals' beliefs about state responsiveness were significantly and positively associated with their sense of collective community efficacy, *b* = .23, 95% CI [.18, .28], *ꞵ* = .22, *p* < .001. Collective community efficacy was also significantly associated with collective action intention, *b* = .15, 95% CI [.10, .21], *ꞵ* = .16, *p* < .001. To test the *catalyst* effect hypothesis, we analysed the indirect effect of state responsiveness on collective action intention via collective community efficacy. The indirect effect was significant, *b* = .04, 95% CI [.02, .05], *ꞵ* = .03, *p* < .001.

Beliefs about state responsiveness were negatively associated with the perceived threat from criminal groups, *b* = −.09, 95% CI [−.13, −.04], *ꞵ* = −.09, *p* < .001. The effect was small but significant. The perceived threat from criminal groups was also significantly associated with collective action intention, *b* = .20, 95% CI [.15, .26], *ꞵ* = .19, *p* < .001. To test the *complacency* effect hypothesis, we analysed the indirect effect of state responsiveness on collective action intention via perceived threat from criminal groups. We found a significant negative indirect effect across nations *b* = −.02, 95% CI [−.03, −.01], *ꞵ* = −.02, *p* = .001.

## DISCUSSION

Despite the importance of civil society's collective engagement against organized crime, few studies have examined people's intentions to mobilize against this societal challenge (Travaglino & Abrams, 2019; Travaglino et al., 2014). In the present research, drawing on a large dataset from eight countries, we investigated for the first time the associations between individuals' perceptions of state responsiveness and their intentions to engage in collective action against organized crime. We proposed two distinct and opposing indirect paths linking state responsiveness to collective action intentions. Specifically, we postulated that the path via heightened collective community efficacy would be linked to an increased willingness to engage in collective action (*Catalyst Indirect Effect*), whereas the path via reduced perceived threat from organized crime would be linked to diminished intentions to engage in collective action (*Complacency Indirect Effects*).

The results provided evidence for the hypothesized indirect effects. First, individuals' perception of state responsiveness was positively associated with collective community efficacy. Community efficacy was, in turn, associated with increased intentions to engage in collective action against criminal groups. This finding indicates that collective community efficacy plays an important role in citizens' intentions to engage in collective mobilization against organized crime.

The results also supported an association between perceived threat and collective mobilization. This finding is consistent with the notion that threat perceptions are linked to collective mobilization (Amel et al., 2017; Kutlaca et al., 2019; Stollberg & Jonas, 2021) as individuals and communities endeavour to restore a sense of security in their surroundings. Interestingly, individuals' beliefs about the responsiveness of institutional authorities were negatively associated with the perceived threat posed by criminal groups (cf. Lin, 2023). This created a small but significant negative indirect effect of state responsiveness on mobilization.

Taken together, the results from a variety of countries in different regions of the world support the idea that individuals' views of state authorities are indirectly associated with their mobilization intentions. The association can be either positive or negative depending on the indirect route. When individuals perceive that institutions and authorities are supportive, this may enhance feelings of empowerment and collective efficacy within local communities. Subsequently, the belief that citizens can collectively address problems and achieve their goals may be associated with a greater likelihood of collective engagement. However, the findings also suggest that state intervention should be carefully calibrated to avoid inducing complacency in individuals by reducing the perception of threat. While more research is needed to confirm the causal links hypothesized, the patterns of associations emerging in the present study suggest that state institutions should aim to foster efficacy while at the same time maintaining a sense of vigilance and urgency about the problem. This balance might contribute to ensure that while citizens feel supported by the authorities, they also remain motivated to take proactive measures in addressing community issues. By better understanding the mechanisms involved in such a balance, policymakers can design interventions that encourage both civic engagement and sustainable social change across various domains, including organized crime. However, these recommendations should be considered independently of any potential contextual effects between countries, as the variability between countries was fully controlled for in the research model of the present study.

### Limitations and future research directions

In the present study, we used country fixed effects in our model to account for individuals' clustering in different countries. Fixed effects are particularly useful when analysing data with few clusters, as they control for between‐cluster variability (Huang, 2016; McNeish, 2023; McNeish & Kelley, 2019). However, a limitation of this approach is that the results cannot be generalized beyond the countries included in the analyses. Additionally, while fixed effects reduce the problem of omitted variables at the country level, they do not allow for the exploration of contextual effects within the model, as they essentially ‘eliminate’ cross‐country variability. Future research should test the current model in a larger sample of countries to investigate its generalizability to different contexts and the potential moderating role of country characteristics. For example, objective indicators of state competency (e.g., Worldwide Governance Indicators) or indices quantifying the influence of organized crime in a country (e.g., Global Organized Crime Index) might be employed to examine how such factors enhance (or weakens) feelings of state responsiveness and contribute to strengthening (or weakening) individuals' and communities' sense of empowerment and perceived threat.

Research involving a larger number of countries and focusing on cross‐country comparisons should carefully consider the issue of measurement invariance of the constructs. In this study, we achieved full configural and metric invariance for all measures. However, full scalar invariance was obtained only for the measure of collective community efficacy, while partial scalar invariance was found for the others. This suggests that, although the measurement scales were generally interpreted similarly across countries, some differences in the baseline levels of the latent constructs existed. Since our primary focus was on the relationships within the mediation model, and we controlled for country‐specific differences through the use of fixed effects, we do not expect these baseline differences to have substantially biased our results. Fixed effects controlled for unobserved heterogeneity at the country level, reducing the risk of bias from cultural or national variations. However, fixed effects cannot eliminate discrepancies in measurement across countries, and we acknowledge this as a limitation of the present research. Future studies should explore further these group‐level variations and consider employing methods such as alignment optimization to enhance cross‐country comparisons (Davidov et al., 2014).

Although our research model and hypotheses were developed based on a review of past research, we acknowledge that our hypotheses and data analysis strategies were not preregistered. However, we have openly shared the data and analysis code used in the present study. Moreover, interpretations of the results should also consider the correlational nature of the findings. The correlational design employed in this research limits our ability to draw causal inferences about the relationships between variables. Future studies should use experimental methods to examine causal links between constructs. Additionally, longitudinal designs could be employed in future work to investigate how the relationships between variables develop over time.

Finally, in this study, we demonstrated a positive association between collective community efficacy and intentions to mobilize against criminal groups. Future research could extend these findings by considering the moderating role of emotions such as hope. For instance, Cohen‐Chen and Van Zomeren (2018) experimentally showed that group efficacy beliefs affect collective action intentions only when change is perceived as possible, which occurs when individuals feel stronger hope. Feelings of hope about change might be especially important in societies where the problem of organized crime has been long‐lasting, such as in Italy.

### Concluding remarks

The present research highlights the complex role of state responsiveness in individuals' intentions to mobilize against collective problems in their communities. State responsiveness can be linked to increased collective mobilization intentions via a stronger sense of empowerment. However, there is also evidence that state responsiveness may be associated with a lower perception of threat, which could, in turn, be linked to reduced mobilization. Policies addressing organized crime may benefit from considering the different ways individuals' views of state authorities can be linked to their collective engagement. Additional research is needed to further explore this topic.

## AUTHOR CONTRIBUTIONS

**Chanki Moon:** Conceptualization; data curation; formal analysis; investigation; methodology; writing – original draft; writing – review and editing. **Giovanni A. Travaglino:** Conceptualization; data curation; formal analysis; investigation; methodology; writing – review and editing. **Alberto Mirisola:** Formal analysis; methodology; writing – review and editing. **Pascal Burgmer:** Methodology; writing – review and editing. **Silvana D'Ottone:** Methodology; writing – review and editing. **Isabella Giammusso:** Methodology; writing – review and editing. **Hirotaka Imada:** Methodology; writing – review and editing. **Kengo Nawata:** Methodology; writing – review and editing. **Miki Ozeki:** Methodology; writing – review and editing.

## FUNDING INFORMATION

This work was supported by the UKRI under Grant ‘Secret Power’ No. EP/X02170X/1 (awarded to GA Travaglino under the European Commission's ‘European Research Council—STG’ Scheme).

## CONFLICT OF INTEREST STATEMENT

The authors declared no potential conflicts of interest regarding the research, authorship and/or publication of this article.

## Supporting information


**Table S1.** Results of Meta Analytical Path Model.
**Table S2.** Results of the indirect effects within the structural equation model separately for each country.

## Data Availability

Data and Code for this article are available at the following link: https://osf.io/hb7yk/

## Footnotes

^1^
The state responsiveness scale's internal reliability was lower in South Korea. Nonetheless, the average inter‐item correlation was .43, an acceptable value for aggregating the items (Clark & Watson, 2019).

^2^
We also tested the catalyst and complacency indirect effects using an alternative analytic approach, specifically a meta‐analytical path model (M. W.‐L. Cheung & Chan, 2005; Jak & Cheung, 2024). This analysis was performed using the metaSEM package in R. The results for the indirect effects were similar to those from the SEM with country‐fixed effects (see Table S1 in the online supplementary material).
