# Supplementary material for: State responsiveness, collective efficacy and threat perception: Catalyst and complacency effects in opposition to crime across eight countries
Source: Br J Soc Psychol. 2024 Dec 11;64(1):e12832. doi: 10.1111/bjso.12832 (PMC11633085; doi:10.1111/bjso.12832)
Supplement: Supplementary file 1 — Table S1. Results of Meta Analytical Path Model. Table S2. Results of the indirect effects within the structural equation model separately for each country. [file BJSO-64-0-s001.docx]

State Responsiveness, Collective Efficacy, and Threat Perception: Catalyst and Complacency Effects in Opposition to Crime Across Eight Countries

**Authors:**

^1^Chanki Moon (corresponding author’s email: [chanki.moon@rhul.ac.uk](mailto:chanki.moon@rhul.ac.uk))

^1^Giovanni A. Travaglino

^2^Alberto Mirisola

^3^Pascal Burgmer

^4^Silvana D'Ottone

^2^Isabella Giammusso

^5^Hirotaka Imada

^6^Kengo Nawata

^7^Miki Ozeki

**Affiliations:**

^1^Institute for the Study of Power, Crime and Society, Department of Law and Criminology, Royal Holloway University of London, UK

^2^Department of Psychology, Educational Science and Human Movement, University of Palermo, 90128, Palermo, Italy

^3^School of Psychology, University of Southampton, Southampton, UK

^4^School of Psychology, Pontificia Universidad Católica de Chile, Chile

^5^Department of Psychology, Royal Holloway University of London, UK

^6^Faculty of Humanities, Fukuoka University, Japan

^7^Faculty of Humanities and Social Sciences, Okayama University, Japan

**Online Supplementary Materials**

- Table S1. Results of Meta Analytical Path Model.
- Table S2. Results of the Indirect effects within the structural equation model separately for each country.

Table S1

Results of Meta Analytical Path Model.

| Direct Paths | Estimate | 95% Confidential Interval | |
| --- | --- | --- | --- |
|  |  | Lower bound | Upper bound |
| SR → CCE | 0.25 | 0.21 | 0.29 |
| SR → PT | -0.10 | -0.14 | -0.06 |
| SR → CAI | 0.03 | -0.01 | 0.07 |
| CCE →CAI | 0.18 | 0.14 | 0.22 |
| PT →CAI | 0.17 | 0.13 | 0.21 |
| Indirect Paths |  |  |  |
| SR → CCE → CAI (Catalyst effect) | 0.05 | 0.03 | 0.06 |
| SR → PT → CAI (Complacency effect) | -0.02 | -0.03 | -0.01 |

*Note*. Standardized coefficients are presented in parentheses. SR = state responsiveness; CCE = collective community efficacy; PT = perceived threat from criminal group; CAI = collective action intention against criminal groups.

Table S2.

Results of the Indirect effects within the structural equation model separately for each country.

|  | UK | | | Italy | | | Germany | | | Korea | | |
| --- | --- | --- | --- | --- | --- | --- | --- | --- | --- | --- | --- | --- |
| Paths | *b*  (ꞵ) | SE | CI_95%_ | *b*  (ꞵ) | SE | CI_95%_ | *b*  (ꞵ) | SE | CI_95%_ | *b*  (ꞵ) | SE | CI_95%_ |
| SR → CCE → CAI  (Catalyst effect) | 0.06  (0.06) | 0.03 | -0.23 to -0.11 | 0.02  (0.02) | 0.02 | -0.01 to 0.06 | 0.05  (0.05) | 0.03 | -0.01, 0.10 | 0.04  (0.03) | 0.03 | -0.02 to 0.10 |
| SR → PT → CAI  (complacency effect) | 0.004  (0.003) | 0.01 | -0.02 to 0.03 | -0.06  (-0.05) | 0.04 | -0.13 to 0.01 | -0.04  (-0.04) | 0.02 | -0.09, 0.01 | -0.04  (-0.03) | 0.02 | -0.08 to 0.01 |
|  | Japan | | | US | | | Chile | | | Colombia | | |
|  | *b*  (ꞵ) | SE | CI_95%_ | *b*  (ꞵ) | SE | CI_95%_ | *b*  (ꞵ) | SE | CI_95%_ | *b*  (ꞵ) | SE | CI_95%_ |
| SR → CCE → CAI  (Catalyst effect) | 0.05  (0.05) | 0.02 | 0.01 to 0.10 | 0.07  (0.07) | 0.03 | 0.02 to 0.12 | 0.01  (0.02) | 0.01 | -0.01 to 0.04 | 0.004  (0.006) | 0.01 | -0.01 to 0.01 |
| SR → PT → CAI  (complacency effect) | -0.02  (-0.02) | 0.02 | -0.06 to 0.01 | 0.02  (0.02) | 0.02 | -0.02 to 0.06 | -0.004  (-0.005) | 0.01 | -0.02 to 0.01 | -0.001  (-0.002) | 0.01 | -0.02 to 0.01 |

*Note*. Standardized coefficients are presented in parentheses. SR = state responsiveness; CCE = collective community efficacy; PT = perceived threat from criminal group; CAI = collective action intention against criminal groups.

It is essential to note that the current study was not designed to focus on testing cross-country differences and the sample sizes in each country are too low for reliable comparisons. Based on simulations presented by Sim et al. (2022), the minimum required sample size needed to test our proposed model in each country (assuming a full mediation) would be *N* = 1,780 (based on small effect size estimates and standardized factor loading greater than .7, two assumptions which are reflected in our results). Additionally, the N = 8 at level 2 (country) is too small to employ multilevel models. Conversely, using the fixed effect in our model enabled us to investigate cross-country averages of the path while employing the entire sample size (*N* = 2,088) and controlling for stable cross-country differences. Although the table above reports the indirect effects for each county (upon request by a reviewer), *these results must be interpreted with caution because this approach has critical limitations for the reasons discussed above*.
